# Revisiting the Corticomotor Plasticity in Low Back Pain: Challenges and Perspectives

**DOI:** 10.3390/healthcare4030067

**Published:** 2016-09-08

**Authors:** Hugo Massé-Alarie, Cyril Schneider

**Affiliations:** 1 Clinical neuroscience and neurostimulation laboratory, Neuroscience Division, Research Center of CHU de Québec, Université Laval, Quebec City, QC G1V 4G2, Canada; 2 Department of Rehabilitation, Faculty of Medicine, Université Laval, Quebec City, QC G1V 4G2, Canada; cyril.schneider@rea.ulaval.ca

**Keywords:** chronic low back pain, brain, plasticity, motor cortex, subgrouping, motor control exercise, neuromodulation, transcranial magnetic stimulation, repetitive peripheral magnetic stimulation, spine

## Abstract

Chronic low back pain (CLBP) is a recurrent debilitating condition that costs billions to society. Refractoriness to conventional treatment, lack of improvement, and associated movement disorders could be related to the extensive brain plasticity present in this condition, especially in the sensorimotor cortices. This narrative review on corticomotor plasticity in CLBP will try to delineate how interventions such as training and neuromodulation can improve the condition. The review recommends subgrouping classification in CLBP owing to brain plasticity markers with a view of better understanding and treating this complex condition.

## 1. Lower Back Pain: A Growing Burden for Society

**Figure 1:**
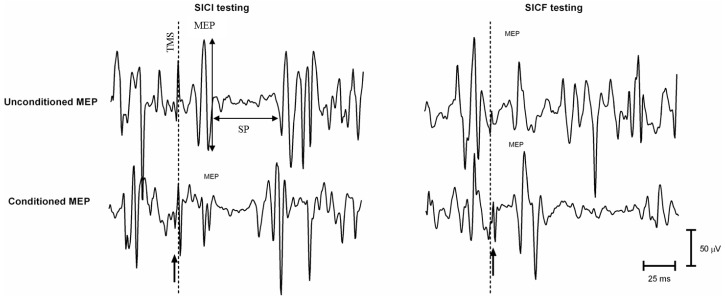
TMS: transcranial magnetic stimulation; SICI: short-interval intracortical inhibition; SICF: short-interval intracortical facilitation; MEP: motor evoked potentials; SP: silent period; small arrow (lower panel): conditioned pulse; dotted line: test pulse.

The important burden of lower back pain (LBP) on healthcare systems can be explained by an extremely high annual prevalence worldwide, i.e., up to 36% of the population [1], which still continues to grow [2]. The Global Burden of Disease mega-study classified LBP as the most debilitating condition among more than 300 diseases for both rich and poor countries [3], and the economic burden increased between 1990 and 2010 [4]. Healthcare costs are among the most expensive in many countries, reaching billions for treating and/or alleviating LBP [5]. Although LBP can decrease after acute episodes, its complete resolution is rare [1,6,7,8,9], and transition to chronic LBP (CLBP) has been reported in up to 12% of people with LBP [10]. Generic administration of conventional treatments (pharmacology, surgery, and physical therapy) has shown no or minimal improvement of pain and disability [11,12]. The pathophysiological mechanisms of CLBP must therefore be better understood in order to identify which therapy is most efficient per individual and thus overcome refractoriness to treatment. Especially, the plasticity of the central nervous system in response to pain (CNS adaptation to pain) represents one of the most important phenomena that could highlight why people with CLBP are poorly responsive to conventional therapies. For instance, transcranial magnetic stimulation (TMS, see Box 1 and Figure 1, [13]) is a widely used technology that permits the investigation of the excitability, functional organization and integrity of the primary motor cortex (M1) that is largely involved in pain processing and motor control. The present narrative review had three main objectives: (i) to report the current knowledge on the changes of M1 and other cortical motor areas in people with CLBP, (ii) to recommend that the research fields of CLBP subgrouping (to reduce heterogeneity of samples studied) and M1 plasticity (biomarkers of brain adaptation to pain) shall be combined to identify new optimal treatment for each patient, and (iii) to present how current interventions such as motor training and neurostimulation techniques impact pain and M1 plasticity [14,15,16,17].

Box 1 **Transcranial magnetic stimulation (TMS).**

TMS represents a painless and non-invasive technique to investigate the function and integrity of the primary motor cortex (M1) and corticospinal pathway. In 1985, Barker et al. published a game-changer paper in the field of clinical neurophysiology, reporting that the induction of a magnetic field over M1 by a coil (where a transient and large electrical current transits from a capacitor system) could depolarize the corticospinal cells. At a sufficient level of intensity, the stimulus produces a muscle response, referred to as motor evoked potential (MEP) recorded by electromyography (EMG) electrodes [18]. MEP latency and amplitude are considered the primary outcomes studied to probe the corticospinal function. Overall, the integrity of corticospinal, intracortical, interregional and interhemispheric connections can now be assessed by the means of different TMS paradigms that are briefly reviewed below.

***Single Pulse TMS***

This paradigm provides at least four outcomes used to test the corticospinal excitability and the functional organization of M1.

The motor threshold (MT) reflects the cortico-cortical excitability of M1 axons, their excitatory contact with the corticospinal neurons, and its initial axon segment [19]. It represents the lowest intensity of stimulation producing an MEP in 50% of TMS trials [20].

The MEP amplitude at supra-threshold intensity (e.g., 110%–120% of motor threshold) represents the excitability of the corticospinal tract. This outcome can be influenced by any change of activity at the cortical or spinal level. The use of pharmacological drugs revealed that the MEP amplitude is regulated by the intertwined activation of excitatory (glutamatergic) and inhibitory (GABAergic) interneurons of M1 [19].

The silent period (SP) is tested in preactivated conditions. SP represents the post-MEP shut-off of EMG activity and its duration over 50–75 ms (0–50 ms = motoneurons after-hyperpolarisaton) probes, most likely the activity of GABA_B_ inhibitory mechanisms of M1 [21].

The M1 mapping is a method to test the functional organization of a muscle representation in M1 and the related corticospinal excitability. TMS at suprathreshold stimulus is applied at multiple sites over M1 and the mean MEP amplitude at each site allows to visually representing the M1 area of the target muscle [22].

***Paired-Pulse TMS***

Two TMS stimuli are elicited over M1 at a given time-interval through the same coil to probe the excitability of M1 inhibition and facilitation circuits.

The paradigm of short-interval intracortical inhibition (SICI) probes the excitability of GABA_A_ inhibitory interneurons surrounding M1 corticospinal cells [19]. A subthreshold conditioning TMS elicited 1–5 ms before a suprathreshhold (test) TMS [23] decreases the amplitude of the conditioned MEP (elicited by the paired-pulse TMS) compared to the amplitude of the test MEP (elicited by the test TMS only).

The paradigm of short-interval intracortical inhibiton (SICF) probes the excitability of a chain of glutamatergic excitatory interneurons connecting M1 corticospinal cells. Two near-threshold TMS stimuli are elicited [24] or a suprathreshold TMS is elicited 1.1–1.5,2.3–2.9 and 4.1–4.4 ms before a near-threshold conditioning TMS [25]. The amplitude of the conditioned MEP is higher than the amplitude of the test MEP. More details can be found elsewhere [19].

***Double-Coil TMS***

This paradigm uses two coils to test the nature (inhibition, facilitation) of other regions’ connectivity with M1 areas. The conditioning TMS coil is positioned, for example, over a premotor or cerebellar area and the test TMS coil over the M1 area of a target muscle. Many studies focused on inter-regional connectivity for the control of hand muscles [26]. This functional connectivity has never been studied for the control of postural muscles (e.g., trunk muscles) in pain-free individuals or in people with LBP.

## 2. Plasticity in M1 and Motor-Related Cerebral Areas

### 2.1. Can M1 Plasticity Explain Motor Impairment in People with CLBP?

Motor control is an important issue in CLBP given that impairment of spine control (and more precisely of trunk abdominal and paravertebral muscles) is deemed to contribute to pain persistence over time [27]. Disorder of spine control in CLBP is the main rationale behind motor control exercises and manual therapy, i.e., interventions used by healthcare professionals to restore an optimal control and mobility of the spine [28,29,30,31]. That is, a large amount of studies have reported that people with CLBP differently plan movement [32,33] and differently react to a postural perturbation [34] as compared to pain-free counterparts. Especially, they present with a later activation of trunk muscle contraction during rapid limb movement, i.e., a delay of the anticipatory postural adjustment (APA) [30], and also less ability to volitionally and specifically contract trunk muscle, abdominal or paravertebral, without recruitment of adjacent muscles [35]. Given the involvement of many cortical structures (M1, supplementary motor area (SMA), cerebellum, basal ganglia, etc.) in APA planning and execution [36], and given that trunk muscles are likely partly controlled by corticospinal pathways [37,38,39,40], it was legitimate to anticipate a link between motor impairment and some (plastic) changes in M1 and other cortical motor areas [41]. This assumption was fostered by a first TMS study that unraveled in LBP a direct relation between M1 functional reorganization (changes of M1 maps) and the delay of trunk muscle activation to control for postural perturbation during focal limb movement [42]. Other TMS studies in CLBP then reported a decrease of M1 excitability [43], changes of M1 area localization for the control of trunk muscles [42,44,45] and a lack of intracortical motor inhibition within M1 circuits [46,47], i.e., the loss of an inherent mechanism of motor preparation [48] and planning [49].

M1 integrates information from adjacent sensorimotor areas (e.g., premotor dorsal and ventral cortices, SMA, cerebellum, basal ganglia, primary sensory cortex (S1), etc.) before launching the motor command towards the spinal motoneurons [50]. Thus, beyond the sole M1 plasticity in CLBP, it is important to understand that many structures can be involved in pain and motor disorders in CLBP. In line with this, studies using neuroimaging techniques (electroencephalography (EEG), functional magnetic resonance imaging (fMRI), etc.) did report changes of grey matter density in various brain structures and impairment of connectivity between these structures [17,51,52,53].

Especially, S1 might be a pivotal stone in the relation between pain and impaired motor control of movement given its substantial role in both the sensory coding of movement and the sensory-discriminative aspects of pain [54,55]. The reciprocal connectivity between S1 and M1 [56] may explain why peripheral inputs (nociceptive, somatosensory) can influence in parallel the plasticity of S1 and of M1 [57,58]. Precisely, in CLBP, S1 grey matter density is different from pain-free couterparts [59], S1 areas receiving information from the trunk are shifted, and connectivity with M1 is impaired [60]. All of these changes likely contribute to the distortion of body image and tactile dysfunction [61,62] but also to the lesser performance in spine motor control by people with CLBP [61]. In addition, abnormal neural processing and connectivity of SMA [52,63] and altered connectivity and change in white matter density of cerebellum [52,64] have been reported in CLBP. These structures are known to be involved in postural control of external perturbations [63] and in APA [36] via transcortical and cerebello-cortical connections with M1 areas. The modifications of cerebellum have been observed in parallel with a slower performance at the sit-to-stand task and an altered proprioceptive integration [52,64].

Interestingly, from a psychological perspective, morphology and connectivity of the brain have also been reported for structures involved in the perception and evaluation of fear, such as amygdala and insula [65,66], these changes having recently been identified as neural correlates of the fear of movement (kinesiophobia) in CLBP [67]. These psychological aspects can alter motor control in CLBP given the correlations found between scores of kinesiophobia or fear-avoidance belief and activation of trunk muscles [47,68,69,70], trunk stiffness [71], and the increased stress on spine structures (spine loading) [72].

**Figure 2:**
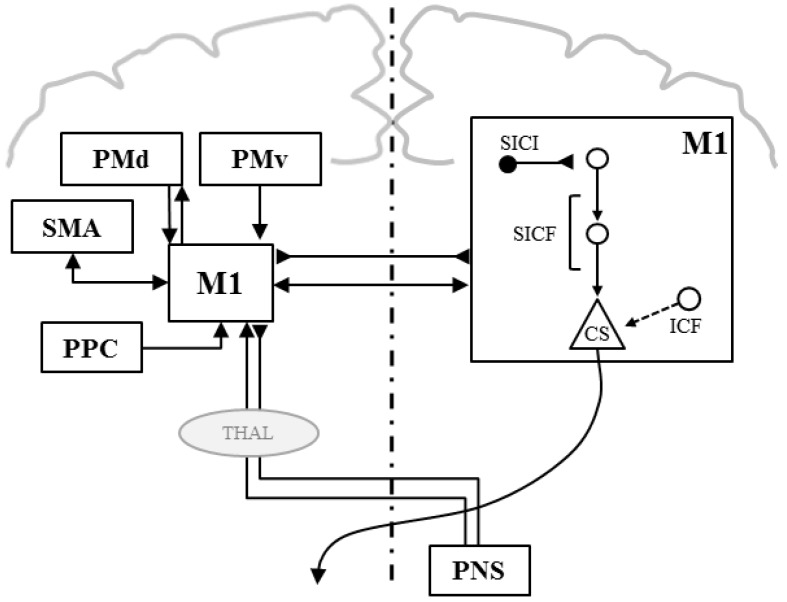
The nature of interregional and interhemispheric connectivity with M1 (left brain) and intracortical connections in M1 (right brain) for hand muscles. M1: primary motor cortex; PMd/v: dorsal/ventral premotor cortex; SMA: supplementary motor area; PPC: parietal posterior cortex; THAL: thalamus; PNS: peripheral nervous system: SICI: short-interval intracortical inhibition; SICF: short-interval intracortical facilitation; arrow: excitatory influence; inverted triangle: inhibitory influence. Adapted with permission from Reis et al. [49].

However, the causal relations in CLBP between pain persistence, motor control and brain plasticity have not been appropriately addressed in the literature with most studies being cross-sectional. However, a few longitudinal studies pointed out that the strength of the connection between mesolimbic and prefrontal area could be an important predictor of pain chronicity [51,73]. Future longitudinal studies in CLBP should thus more thoroughly test the role of M1 and other motor control-related cerebral plasticity in the persistence of LBP. For instance, the integrity of M1 functional interregional connectivity with SMA and cerebellum and the remote inhibitory and excitatory influence of these structures on M1 excitability could be assessed by means of TMS paradigms using two coils (see Figure 2). These paradigms might help unravel the mechanisms underlying the impaired corticomotor control of trunk muscles in people with CLBP and the transition from acute to chronic pain.

### 2.2. Discrepancies between TMS Studies in CLBP: How to Reconcile the Controversial Results?

A closer look at the studies that pointed out differences of M1 maps, excitability and function between people with CLBP and pain-free subjects [42,43,44,45,47,74,75] reveals important discrepancies between the results. For instance, Strutton et al. reported that people with CLBP presented with a lower M1 excitability (measured by motor threshold—MT) and a decreased GABA (γ-aminobutyric acid) inhibition (measured by the silent period duration, see Box 1) [34]. However, these findings have not been yet reproduced [46,47,76]. In addition, our recent TMS studies in CLBP did not detect any difference of MT or SP duration but rather a reduction of GABA_A_ short-interval intracortical inhibition (SICI) in the M1 area of internal oblique/transversus abdominis muscles [47] and superficial multifidus [46]. Our more recent works even conversely showed that a subgroup of people with right-sided CLBP presented with a higher M1 excitability (lower MT) compared to pain-free counterparts (Massé-Alarie et al., *in revisions* [77]). In the same vein, Hodges’ group studies in CLBP showed the impaired organization of trunk muscles M1 areas (for erector spinae [44] and transversus abdominis muscles [42]), M1 plasticity being more important in a subgroup of people with severe CLBP (>5 on numerical rating scale) than moderate and mild CLBP [45] and upper CLBP relating to smaller map volumes, thus likely a decrease of corticospinal excitability [45].

Therefore, even though most of these studies reported changes in one or more TMS outcomes, no one has been replicated yet. In line with Schabrun et al. (2015) [36] and our unpublished data [77], it is proposed that the heterogeneity of the nonspecific CLBP population tested has hindered specific differences of M1 function in literature, some subgroups presenting with M1 plasticity and others not. The next section briefly reviews some classification known in LBP and how this could be useful in TMS and neuroimaging studies to detect changes specific to subgrouping.

## 3. Subgrouping of CLBP in Neuroplasticity Studies

The inherent heterogeneity of CLBP population affects the understanding of plastic phenomena and thus hinders the knowledge of the actual clinical impact of novel and conventional therapies. Despite the validation of several models of classification in the last two decades [78,79], only a few neuroimaging or TMS studies have used subgrouping of people with CLBP. A better delineation of the link between characteristics of CLBP (subgrouping) and components of brain plasticity, and a better understanding of the significance of this plasticity in pain processing and disability, are, therefore, current challenges to better managing CLBP and guiding rehabilitation.

### 3.1. Subgroups Based on the Nature of CLBP

Smart et al. (2010) proposed classifying people with CLBP into three subgroups relative to the nature of pain, i.e., nociceptive pain (peripheral structure injury, 55% of CLBP population), neuropathic (nerve lesion, 22%) or “central sensitization” (characterized by a diffuse disproportioned pain and exaggerated response of CNS to sensory inputs, 23%, see Box 2 [80,81,82]). Brain plasticity has, however, never been tested as a function of the nature of CLBP. Interestingly, TMS studies on other pain conditions showed that changes of M1 function and excitability were more important in people with neuropathic pain (radiculopathy [83]) or “central sensitization” (fibromyalgia or complex regional pain syndrome (CRPS) [84,85,86]) than in people suffering from specific nociceptive pain (finger osteoarthrosis) [87]. For instance, people suffering from CRPS, fibromyalgia and people with neuropathic pain were all tested with a reduction of the level of inhibition (measured by SICI, see Box 1) [84,85,86,88]. Thus, testing nonspecific CLBP, i.e., a heterogeneous population, may explain the inconsistent, even controversial, findings on M1 excitability and function across studies. Future studies should indeed enroll subgroups of patients with CLBP in order to better tackle the link of nociceptive, neuropathic or centrally sensitized CLBP with the plasticity of M1 circuits that likely contributes to pain and motor impairment. If brain plasticity, as tested by TMS of M1, is different between subgroups of CLBP, then TMS will be a useful tool to identify, for example, the sensorimotor mechanisms impaired in people with “central sensitization”, and thus will help to manage each person with the most appropriate treatment (e.g., with neuromodulation technique, see Section 4). Some challenges have to be overcome, however, in order to utilize TMS for that purpose. First, normative values of the M1 control of trunk muscles, for example, have to be determined in order to assess any M1 dysfunction in CLBP. This will be challenging given the important variability in TMS outcomes. In addition, TMS markers of M1 function, such as M1 inhibition and facilitation (SICI, ICF), are changed in various pathological conditions (e.g., psychiatric or neurological diseases [89,90], likely because of the multiple cerebral and peripheral influences onto M1 circuits [26] (Figure 2). Thus, and as already mentioned, future longitudinal studies should use double-coil TMS paradigms or TMS tools combined with neuroimaging techniques to unravel the faulty mechanisms (structures, connectivity) in specific subgroups of CLBP.

Box 2 **Central sensitization**

“Central sensitization” is defined as “an amplification of neural signaling within the CNS that elicits pain hypersensitivity” [91]. The term was introduced to describe changes found at the spinal cord level ([92]), i.e., a post-injury amplification of the peripheral nociceptive signal by CNS hyperexcitability. “Central sensitization” implies that innocuous inputs from the periphery might be perceived as painful if the “pain pathway” is facilitated either at the spinal or cerebral level. By extension, the hyperalgesia documented in subgroups of people with CLBP [93], in addition to the alteration of brain connectivity and morphology (e.g., dorsolateral prefrontal cortex [14,15,16,17], periaqueductal grey matter), could be interpreted as “central sensitization” because it likely reflects the alteration of pain modulation by descending pathways that might favour pain persistence.

The term “central sensitization” is used in clinical practice to describe a subgroup of people with specific clinical characteristics [80]. In CLBP, this corresponds to three criteria: (i) disproportionate pain, (ii) neuroanatomically illogical pain pattern, and (iii) hypersensitivity of senses unrelated to the musculoskeletal system [80]. These criteria can delineate people with “central sensitization” patterns from people with nociceptive and neuropathic pain.

Nociceptive pain refers to pain coming from the activation of nociceptors of non-neural tissue in response to noxious chemical, mechanical or thermal stimuli [80,81] (e.g., the activation of the nociceptors in lumbar ligaments, thoraco-lumbar fascia or zygapophyseal joints). Neuropathic pain refers to pain secondary to a disease or a lesion of the somatosensory nervous system [80] (e.g., LBP associated with lumbar radiculopathy).

In recent studies, people with CLBP are classified in three different subgroups owing to the nature of pain: nociceptive, neuropathic or “central sensitization” [80,81,82,94,95,96,97,98]. Please refer to the clinical guideline proposed by Nijs et al. (2015) for additional details about this classification [80].

### 3.2. Subgroups Based on the Nociceptive Somatosensory Processing: Mechanical vs. Non-Mechanical CLBP

The O’Sullivan’s group published a series of studies lately that insisted further on the need to monitor heterogeneity of samples in CLBP research [93,99,100]. Precisely, nociceptive and somatosensory processing appeared to be significantly different in people with a mechanical CLBP (i.e., pain increased by a specific movement, posture or activity) as compared to people with a non-mechanical CLBP (i.e., spontaneous pain not related to a specific movement, posture or activity). Only people with non-mechanical CLBP presented with a decrease in cold pain threshold as compared to pain-free subjects [99]. In addition, differences of somatosensory nociceptive processing were detected in a large CLBP cohort where three subgroups were differentiated, but only two of them behaved differently than the pain-free subjects [93]. This mechanical vs. non-mechanical CLBP classification could also help to discriminate plastic changes in the brain. Indeed, given tight functional connections between S1 and M1 areas [101], it is likely that subgroups presenting with impaired somatosensory nociceptive processing and S1 plasticity will also undergo plasticity of M1 areas.

### 3.3. Subgroups Bbased on Movement Disorders

Many classifications based on the type of movement disorders and on movements generating pain in LBP have been validated in the last years [78]. For instance, people with CLBP predominantly triggered during lumbar extension did present with an increase of paravertebral muscles activity in sitting [102] and during forward bending [103] as compared to pain-free participants, whereas people with CLBP triggered primarily during flexion did not. These differences were masked when the two CLBP subgroups were considered as one CLBP group [103]. Such classification should be tested to detect whether M1 plasticity is specific to movement disorders in CLBP. This will help to identify biomarkers of brain function and excitability that will be useful in the management of the clinical outcomes specific to each subgroup. The next section will present how different types of interventions such as motor training and neuromodulation technologies might impact pain and the brain.

## 4. Interventions Targeting M1 Plasticity

### 4.1. Learning-Dependent Plasticity in CLBP: How Motor Training Impacts M1?

Studies revealing the extensive brain plasticity in CLBP contribute to a better understanding of the physiopathology mechanisms present in pain pathologies. This ought to guide the development of therapies that will better cope with brain adaptation. In other words, the induction of plasticity that promotes the function and reduces pain (positive plasticity) should become the rule in pain rehabilitation. For instance, training the tactile acuity normalized S1 maps and in parallel improved sensory integration in CLBP [104,105]. The same is true for motor training in CLBP since the practice of isometric activation of the trunk muscles normalized M1 maps [106] and influenced the intracortical inhibition required for planning the action [47] and the corticospinal excitability [107]. In fact, the induction of a positive plasticity (favoring functional recovery) requires a task-oriented practice, i.e., the repetition of a specific task, whose complexity is increased with improvement of performance over the sessions and attention/motivation of the trainee [108]. Usually, the improvement of a motor skill is accompanied by changes of M1 protein synthesis (for instance, tyrosine kinase), synaptogenesis and reorganization of S1 and M1 maps [108]. In addition, a local release from GABAergic inhibition in M1 areas recruited by task-specific training and an increase of corticospinal excitability related to the muscles engaged in the task are usually observed in the minutes following motor learning [109,110,111,112]. These plastic mechanisms of motor learning belong to the LTP-like phenomenon that strengthens the synapses efficacy and includes the activation of post-synaptic NMDA (N-methyl-D-aspartate) receptors and the increase of AMPA (α-amino-3-hydroxy-5-methyl-4-isoxazolepropionic acid) receptor density [110,111]. However, the question remains whether these learning-related mechanisms of plasticity do properly work in the presence of pain.

Motor training influences brain plasticity and this contributes to motor learning. For example, motor control exercise (MCE) is used in CLBP physical therapy to restore the proper balance of activation between trunk muscles (usually increasing deep muscle activation and reducing superficial) and, eventually, to transfer this re-learned muscle coordination in functional tasks [30]. In line with this, it was shown that MCE could normalize M1 maps in people with LBP [106], downregulate the exaggerated corticospinal excitability related to superficial paravertebral muscles and upregulate the missing intracortical inhibition needed for motor planning [107]. This influence of MCE on corticomotor plasticity is thought to promote the postural function of the trunk muscles (e.g., APA) in CLBP, thus normalizing the postural control of the spine [106]. However, despite these intertwined cerebral and functional changes, meta-analyses and systematic reviews underlined that exercise therapy and motor control training worked poorly on pain and disability in CLBP [11,113,114], and no therapy seems more effective than another [114]. In addition to subgrouping (focus on specific subgroups of CLBP) and/or patient-oriented training (personalized care), as discussed above, an increasing number of studies investigated whether new techniques of modulation of CNS excitability could further influence pain intensity, brain plasticity, and motor disorders in CLBP beyond the gains already reached by conventional therapies alone.

### 4.2. Noninvasive and Painless Neuromodulation in CLBP

Noninvasive and painless neuromodulation in CLBP is a new area of research to influence CNS plasticity directly by stimulation of brain circuits (central stimulation: top-down mechanisms involved) or indirectly by stimulation of the lower back (peripheral stimulation: bottom-up). This influence on CNS plasticity ought to help decrease pain, improve sensory integration, and normalize the sensorimotor control of posture and movement (all being intertwined in the management of CLBP). Neuromodulation techniques that are known to increase the corticospinal excitability (e.g., high-frequency repetitive transcranial magnetic stimulation (rTMS), intermittent theta-burst stimulation (iTBS) or anodal transcranial direct current stimulation (tDCS)) share similar mechanisms of neuroplasticity with motor learning, i.e., LTP-like phenomenon with changes of M1 GABAergic inhibition and NMDA-dependent facilitation (for an extensive review on plastic mechanisms following noninvasive neurostimulation, see [19]). Thus, these mechanisms influenced by neuromodulation could prime the brain before beginning a conventional therapy, and this may increase gains beyond those reached by each intervention alone. The next sections present the literature on central and peripheral neurostimulation and their combination with therapy in CLBP to decrease pain and promote the function.

### 4.3. Central Stimulation

The mechanisms’ underlying pain decrease following M1 stimulation could rely on the activation of thalamus by cortico-thalamic projections [115], on the inhibition of spinal cord circuits (likely at the dorsal horn) by corticospinal modulatory projections, and on the activation of µ-opioid receptors [116]. Central neurostimulation has been used in research for chronic pain conditions like CRPS, neuropathic pain and fibromyalgia [116,117]. However, only a few studies used rTMS and tDCS in people with CLBP, and evidence of effectiveness is lacking. For example, one study showed that one session of rTMS over M1 improved CLBP intensity and the cold/heat pain threshold [118], but no study has ever tested longer-lasting after-effects following multiple sessions of rTMS [116]. In addition, two recent randomized double-blind designed studies reported that multiple sessions of tDCS over M1 did not improve CLBP [119,120], and one experimental study did not report immediate impact on pain threshold [121]. Thus, due to scarce data published on that topic, there is no clear evidence that central stimulation impacts pain or disability in CLBP.

### 4.4. Peripheral Stimulation

Repetitive magnetic stimulation over muscles, nerves or spinal roots (RPMS) is used in exploratory research to improve motor impairments in brain-injured people [122,123]. The rationale for using RPMS is based on the production of contractions that send massive flows of ascending movement-related proprioceptive information to the brain and influence M1 excitability via thalamo-cortical (lemniscal) and spino-cerebellar pathways [124]. It has been shown that this synchronizes the activity of the fronto-parietal networks involved in motor planning [125]. RPMS is a novel, not yet evidence-based, but promising experimental approach in people living with chronic pain and presenting with motor disability. One study showed that a single session of RPMS applied over the lumbar spine in people with CLBP could decrease pain and the after-effects persisted for least four days [126]. In addition, RPMS applied over the deep abdominal muscles not only reduced CLBP and improved postural control (APA becoming earlier) but also reactivated proper mechanisms of M1 intracortical inhibition [127], which was shown to be missing or lower in people with CLBP [47]. Altogether, these findings emphasize the potential of this novel approach to act on brain plasticity for decreasing pain and improving motor control. Further investigations are warranted to better understand the link between RPMS and brain adaptation and to determine whether RPMS activates the different components of the endogenous pain modulation system.

### 4.5. Combination of Interventions

The combination of two interventions that influence brain plasticity could impact pain intensity more than each intervention used separately. This was tested in CLBP by tDCS of M1 combined with peripheral electrical stimulation over paravertebral muscles [121]. The authors showed a reduction of pain that was accompanied by M1 reorganization and by improvements of forward bending, pressure pain threshold, and two-point discrimination. However, these after-effects did not last after the end of stimulation [128]. Short-lasting effects could be related to the fact that, in the absence of a lesion, brain circuits can adapt rapidly to any change of activation and could re-balance the synaptic excitability back to physiological ranges of homeostasis: this phenomenon is referred to as metaplasticity [129]. Metaplasticity implies that all increases or decreases of excitability will eventually return to baseline unless repeated over multiple sessions or combined with other therapies, such as motor training that favors similar mechanisms of synaptic plasticity [130]. Changes of M1 excitability following neurostimulation could indeed open a “therapeutic” window during which a task-oriented practice is easier, and, in turn, makes plastic changes more persistent, thus facilitating learning and retention [130], therefore improving motor control and pain over a longer period of time. This was tested by two original studies that combined RPMS with motor control training in people with CLBP in a single session for deep abdominal muscles [127] and over one week for the paravertebral multifides muscles (Massé-Alarie et al., *in revisions* [131]). It was shown that the combination decreased pain more than training alone and that one-week of training induced the normalization of superficial multifides activation in parallel with up regulation of M1 facilitation mechanisms (Massé-Alarie et al., *in revisions* [131]).

Combining neurostimulation with motor training could act at the spinal, brainstem and cerebral levels of the endogenous pain modulation system to re-balance the activity of cerebral networks and areas that do not work properly in CLBP. This may provide gains in pain and disability beyond those already reached by conventional treatments. Therefore, priming the brain with neuromodulation techniques to enlarge the after-effects of conventional therapy [132] represents a research field that ought to be pursued in CLBP.

## 5. How Can Neuroplasticity Studies Better Reduce Pain and Disability in CLBP?

### 5.1. Identifying Brain Biomarkers in CLBP

#### 5.1.1. “Central Sensitization” or Non-Mechanical CLBP

Neuroimaging and TMS studies on neuroplasticity in CLBP can help determine biomarkers that will contrast between people with “central sensitization” (see Box 2) and people with nociceptive pain. These biomarkers may inform on unsuspected impaired function, thus easing the adaptation of the therapeutic approach. For example, “central sensitization” and non-mechanical CLBP that share a common definition (pain not related to a specific spine movement, posture and activity) may be less responsive to conventional motor control exercises, stabilization or passive mobility (e.g., manual therapy or stretching) because of somatosensory nociceptive processing impairments [99] and extensive changes of M1 maps and excitability [84,85,86]. That said, it was shown that the neural connections between medial PFC (mPFC) and NAc were stronger in people with persistent LBP (less than 20% improvement at one year after the first LBP episode) [51]. These people with central sensitization reported higher scores for affective dimension of CLBP (i.e., the pain was considered more threatening), and this is in line with the fact that stronger mPFC-NAc connections could induce negative pain conditioning with aversive or fear-related behavior [51]. In order to influence such behaviors related to pain-related plasticity, novel approaches targeting psychological factors were proposed, such as pain conditioning extinction [133], pain neuroscience education [134], motor control exercise [94] and cognitive-based intervention [135]. These approaches tended to address the psychosocial risk factors associated with CLBP (especially for people with “central sensitization”) [6,80] and took into account that the activation of sensory-discriminative brain areas in subacute pain was switched to the activation of emotional areas during the transition to chronic pain [136]. As a matter of fact, people identified with specific biomarkers of brain plasticity (for instance, a stronger connection between mPFC and NAc) could be subclassified according to their clinical characteristics related to plasticity (for instance, a high score for affective dimension of pain at the McGill Pain Questionnaire) and might be better responders to cognitive-related therapy targeting psychosocial factors than to motor control exercises or to neuromodulation of mPFC to downregulate the facilitation of NAc. Future investigations are warranted to test whether such specific approaches dedicated to people with strong mPFC-NAc (or high affective dimension of pain) better impacts pain persistence. It is also questioned whether neuromodulation of DLPFC could be an interesting adjuvant to cognitive-related therapies in people with important psychosocial factors and central sensitization. Indeed, DLPFC is often targeted with rTMS in chronic pain condition [116], it is implied in endogenous pain system [137,138] and its function is altered in CLBP [15]). Of note, given that M1 (opiate system) and DLPFC (non-opiate system) [116] can have a different influence on CLBP, the choice of stimulating one or the other in combination with a therapy may depend on the patient’s characteristics and biomarkers of neuroplasticity and underlying mechanisms have still to be studied.

#### 5.1.2. Nociceptive or Mechanical CLBP

People who present nociceptive or mechanical CLBP can be divided into subgroups according to movement disorders and thus be treated accordingly. For instance, it was shown that people with a treatment adapted to their clinical profile (according to the Classification Based Cognitive Functional Therapy (CB-CFT) [139]) had larger decrease of pain than people treated with conventional exercise therapy combined with manual therapy [140]. The same results were reproduced in subacute LBP (CB-CFT was more effective than “general exercise”), but with the exclusion of people with high psychosocial risk factors (fear-avoidance, kinesiophobia, depression) and with poor scores on the Motor Control Abilities Questionnaire (MCAQ, which identifies people unable to learn motor control exercise) [88]. The authors suggested that the improvement in the CB-CFT group in their study was actually due to the exclusion of people with poor MCAQ scores. In fact, a lower capacity of motor learning has already been associated with a polymorphism of brain-derived neurotrophic factor (BDNF) [141]. More precisely, these authors showed that the increase of M1 excitability following training, thus usually favoring learning, was reduced in healthy subjects with BDNF polymorphism compared to healthy subjects without. Thus, people with CLBP could also be classified owing to their BDNF polymorphism (blood sampling) or, more conveniently, owing to their responsiveness to a complex motor learning task (increase or not of M1 excitability as tested by TMS), in order to detect rapidly those who might better respond to motor training. In support, we showed that training at isometric activation of the multifides muscles could influence the corticospinal excitability and the intracortical inhibition of M1 (SICI) related to these muscles [107]. Future studies should test whether such changes after one training session (biomarkers of learning-related plasticity) are correlated with MCAQ scores and thus could be useful predictors to identify people who might be responsive to motor control training (i.e., significant increase of M1 excitability, high MCAQ scores) or not (i.e., small or no change of M1 excitability, low MCAQ scores). In addition, peripheral neurostimulation could be an efficient adjuvant in people with CLBP to influence brain plasticity and improve motor learning and pain beyond gains already reached by more conventional therapies (Massé-Alarie et al. *in revisions* [131]). This work denoted that larger improvements were obtained after one week of training in people who presented with lower M1 excitability at baseline. It will thus be important to understand the clinical significance of a low vs. a high M1 excitability in CLBP at enrollment for better identifying people who will be responsive, for example, to peripheral neurostimulation and motor training.

## 6. Conclusions: Avoiding *One Size Fits All* Treatments to M1 Plasticity in CLBP

The extensive brain plasticity in chronic pain has been depicted in the last years with a view of tackling the cortical processes under the clinical characteristics and making recommendations of more efficient interventions [135,142]. Thus, a better understanding of the neurophysiology of pain, and, more precisely, the physiopathology of CLBP (in the absence of any peripheral lesion) did revolutionize the way chronic pain was understood and people with CLBP were managed (integration of the psychological, environmental and social factors, in addition to conventional therapies). However, careful examination of neuroplasticity studies revealed that some CLBP subgroups did not present changes of M1 function, maps or excitability [45] and that literature discrepancies hinder the comprehension, e.g., of whether grey matter density is increased or decreased in target structures [15]. In addition, larger plastic changes of M1 areas were detected for people with “central sensitization” and in relation to psychosocial factors, but this subgroup represents 23% of people with CLBP. Thus, the sole cognitive-based intervention may not be efficient for most patients and motor control disorders have to be considered. Avoiding amalgam for treating CLBP is necessary to better understand and treat this condition appropriately in each individual in consideration of M1 plasticity, motor impairments (posture, movement), psychological issues and social characteristics. Thus, a thorough evaluation of the initial condition of the patients will help personalize treatment owing to clinical characteristics, although it remains challenging to get a clear difference between subgroups (since all pain is in the brain, nociceptive pain likely embedded a certain component of “central sensitization” [80]). Future studies in CLBP will have to consider the recruitment of subgroups of patients in order to identify specific biomarkers of brain plasticity and motor disorders, thus markers of responsiveness to approaches based on individuals’ clinical profile, including peripheral or central neurostimulation as adjuvants to more conventional treatments. The development of such new guidelines, along with the one published in Nijs et al. [80], and integrating the diversity of people with CLBP in relation to clinical features, biomarkers of neuroplasticity, and motor disorders is warranted for the researchers to better test new interventions and for the clinicians to better cope with this condition and decrease the societal burden of CLBP.
